# Dr. Edward Meany

**Published:** 1912-01

**Authors:** 


Dr. Edward Meany, University of Buffalo, 1887, died at Ithaca, November 21, of cerebral haemmorhage, aged 46. He was the first Health Officer of Ithaca and a former president of the Tompkins Co. Medical Society.

